# Patterns of variation in equine strongyle community structure across age groups and gut compartments

**DOI:** 10.1186/s13071-022-05645-5

**Published:** 2023-02-11

**Authors:** Michel Boisseau, Núria Mach, Marta Basiaga, Tetiana Kuzmina, Claire Laugier, Guillaume Sallé

**Affiliations:** 1 INRE, ISP, Université de Tours, Nouzilly, France; 2 IHAP, INRAE, ENVT, Université de Toulouse, Toulouse, France; 3 Department of Zoology and Animal Welfare, Faculty of Animal Science, University of Agriculture in Kraków, 24/28 Mickiewicza Av., 30-059 Cracow, Poland; 4 Department of Parasitology I.I. Schmalhausen Institute of Zoology, National Academy of Sciences (NAS) of Ukraine, Kiev, Ukraine; 5 Institute of Parasitology, Slovak Academy of Sciences, Hlinkova 3, 040 01 Kosice, Slovak Republic; 6 Conseil Général de l’Alimentation, de l’Agriculture et Des Espaces Ruraux, Ministère de l’Agriculture et de l’Alimentation, Paris, France

**Keywords:** Horse, Cyathostomin, Diversity, Species network, Nematode, Co-occurrence, Parasite

## Abstract

**Background:**

Equine strongyles encompass more than 64 species of nematode worms that are responsible for growth retardation and the death of animals. The factors underpinning variation in the structure of the equine strongyle community remain unknown.

**Methods:**

Using horse-based strongyle community data collected after horse deworming (48 horses in Poland, 197 horses in Ukraine), we regressed species richness and the Gini-Simpson index upon the horse’s age, faecal egg count, sex and operation of origin. Using the Ukrainian observations, we applied a hierarchical diversity partitioning framework to estimate how communities were remodelled across operations, age groups and horses. Lastly, strongyle species counts collected after necropsy (46 horses in France, 150 in Australia) were considered for analysis of their co-occurrences across intestinal compartments using a joint species distribution modelling approach.

**Results:**

First, inter-operation variation accounted for > 45% of the variance in species richness or the Gini-Simpson index (which relates to species dominance in communities). Species richness decreased with horse’s age (*P* = 0.01) and showed a mild increase with parasite egg excretion (*P* < 0.1), but the Gini-Simpson index was neither associated with parasite egg excretion (*P* = 0.8) nor with horse age (*P* = 0.37). Second, within-host diversity represented half of the overall diversity across Ukrainian operations. While this is expected to erase species diversity across communities, community dissimilarity between horse age classes was the second most important contributor to overall diversity (25.8%). Third, analysis of species abundance data quantified at necropsy defined a network of positive co-occurrences between the four most prevalent strongyle genera. This pattern was common to necropsies performed in France and Australia.

**Conclusions:**

Taken together, these results show a pattern of β-diversity maintenance across age classes combined with positive co-occurrences that might be grounded by priority effects between the major species.

**Graphical Abstract:**

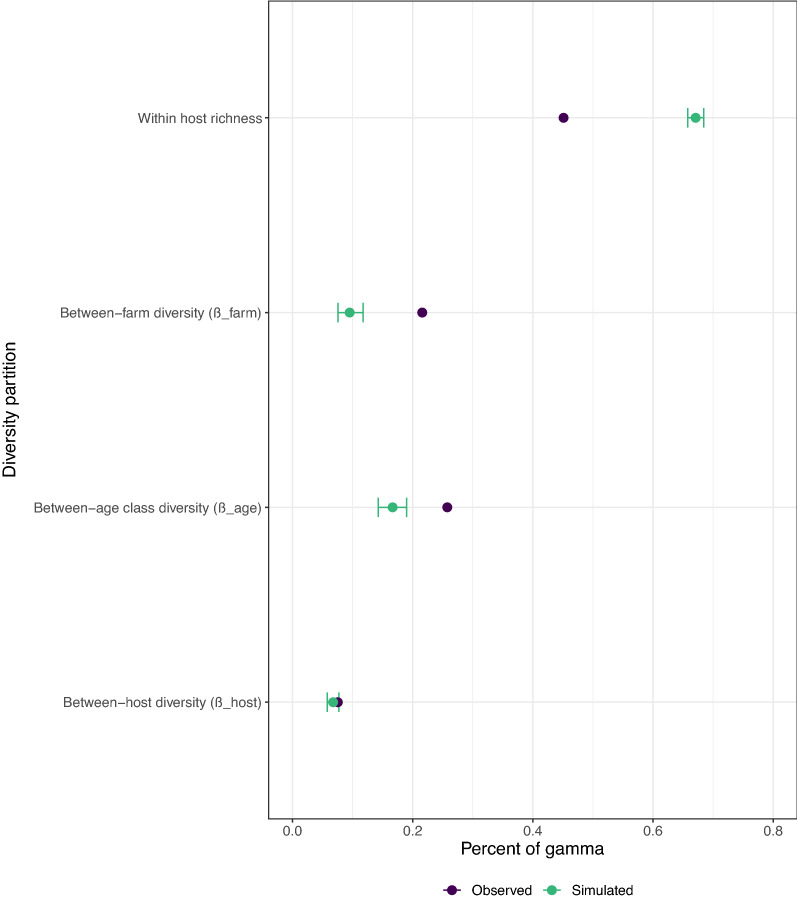

**Supplementary Information:**

The online version contains supplementary material available at 10.1186/s13071-022-05645-5.

## Background

The worldwide accumulation of anthelmintic drug failures against equine strongyles threatens the sustainability of current control strategies. These parasitic nematodes form a complex assemblage that establishes in their host hindgut, leading to nutrient spoliation, poor growth and weight loss [1]. Under yet-to-be resolved circumstances, the massive sudden emergence of developing larval stages can lead to a cyathostominosis syndrome, characterized by protein-losing enteropathy, abdominal pain and eventually the death of horses [1, 2].

Equine strongyles encompass more than 64 species that are responsible for growth retardation and the death of animals [3]. Investigation of regional patterns of equine strongyle community structure has consistently isolated a core of ten to twelve species dominating the overall community across countries [4, 5]. Recent meta-analyses have also supported this consistency across a wide range of geoclimatic conditions [6].

At the horse level, significant variation in strongyle community occurs between sexes [4, 7], and several strands of evidence have also emerged to support strongyle community remodelling as horses age. First, finds from early artificial infection experiments highlighted differential prepatent periods between cyathostomin species [8, 9] that may contribute to variation across age groups. Second, differential relative abundances of cyathostomin species across the range of horse ages were recorded in a regional abattoir in Victoria state, Australia [4]. The most recent investigation of the development of strongyle communities across age groups was performed on horses from Ukraine operations and suggested significant remodelling of their diversity as horses age [10]. However, none of the published studies quantified the relative contribution of age to the dissimilarity found between strongyle communities across horses.

In addition, individual faecal egg count (FEC) excretion has traditionally been used as a marker on which to base decisions of horse treatment. FEC correlates poorly to the number of worms [11–14], but it varies significantly across individuals, and a significant share of this variation is underpinned by an heritable component [15–17]. However, the relative contribution of the strongyle community structure and diversity to this trait remains unknown, although strongyle species number is significantly associated with FEC [10] and higher infection intensity is associated with more diverse strongyle communities [18].

Last, co-occurrence patterns between equine strongylid species remain undefined, with contradictory findings reported [7, 19]. In an abattoir survey conducted in Italy, only positive co-occurrences were found, possibly reflecting the low worm burden observed in the sampled horses [19]. Similarly, most of the correlations estimated from species counts obtained from dewormed horses in Poland were positive, although negative correlations were found between the *Coronocyclus* species [7]. This is despite observations that some species preferentially establish in different gut compartments [4, 5, 20]. It is therefore unclear whether negative interactions between particular species may underpin the dissimilarity between strongyle communities.

Building on published strongyle community data obtained after horse necropsy [4, 18] or deworming [7, 10] in Europe and Australia, this study aims to define additional insights on equine strongyle communities.

## Methods

All statistical analyses were performed using R software v4.0.2 [21].

### Selection and use of datasets

The analyses presented herein were run on horse-based strongyle community data on 441 horses that were obtained from the original protocols of four previously published studies [4, 7, 10, 18]. The selection of studies was as follows. In the frame of a wider meta-analysis [22], we identified 34 papers presenting cyathostomin abundance or prevalence data. To be able to contact the authors and access individual observations and to sample sufficient diversity from the data, we then restricted the datasets to papers published no more than 25 years ago and studies comprising at least 40 horses. This approach retained studies performed in Europe (Table 1), including necropsies performed in Normandy [18] and worm collections after the deworming of horses in Poland [7] and Ukraine [10]. Another dataset remained inaccessible [20].

**Table 1:** Overview of the datasets considered in this study and the analyses performed

Country	Collection method	Sample size (*n*)	Year published	Analyses performed	References
Australia	Necropsy	150	1995	Strongyle species co-occurrence (validation set)	[6]
France	Necropsy	46	2002	Strongyle species co-occurrence (test set)	[19]
Poland	Deworming	48	2018	Relationship between community diversity and FEC	[8]
Ukraine	Deworming	197	2016	Relationship between community diversity and FEC Diversity partitioning	[11]

For each analysis presented in this paper, the corresponding dataset is listed with its original reference and associated metadata

*FEC* Fecael egg count

Our first step was to identify papers associating FEC data and strongyle species counts [7, 10] in order to study the relationship between FEC and species diversity (Table 1). Second, the unique hierarchical nature of the Ukrainian dataset [10] was leveraged to apply a diversity partitioning approach across multiple scales, including horse age groups. (Table 1). Lastly, co-occurrence between strongyle species was investigated from the necropsy data collected in Normandy. To cross-validate the co-occurrence pattern found in this dataset, we looked for independent data from a geographically disconnected area. The Australian dataset by Bucknell et al. [4] was identified from the aforementioned meta-analysis, and the data were obtained from Ian Beveridge (personal communication). However, no metadata were available, and these observations were used only to validate the broad patterns found in the Normandy data.

### Considered diversity indexes

Strongyle community diversity can be described at a regional scale (γ-diversity), such as, for example, a country. This overall diversity can be decomposed into α-diversity, i.e. the diversity within a sampling unit such as a single horse, and β-diversity, which accounts for rearrangements occurring between communities [23, 24]. Species richness is the simplest estimator of α-diversity. A more complex index, such as the Gini-Simpson index (defined as 1 – *p*_*k*_^2^, where *p*_*k*_^*2*^ is the relative abundance of species *k*) corresponds to the probability that two worms chosen at random from a community belong to different species [25, 26]. This index therefore relates to species dominance and is estimated using the ‘diversity’ function of the vegan package [27].

### Relationship between strongyle community diversity and corresponding horse FEC

To evaluate how strongyle community diversity could relate to measured FEC in their host, we first estimated Spearman’s correlations between FEC and species richness, or between FEC and the Gini-Simpson index. This analysis was implemented on the Ukrainian and Polish horses independently but was not performed on the necropsy data as FEC measures were not available for the latter data. To further establish how horse FEC may contribute to species richness and the Gini-Simpson index, both variables were regressed independently upon some effects of interest using the following linear mixed model with the lme4 package [28]:

$${Y}_{ijk}\sim {log(FEC+50)}_{i} + {Age }_{i}+{Sex }_{i,j}+{ Farm }_{i,k},$$

Where *Y*_*i,j,k,l*_ refers to the strongyle community species richness or Gini-Simpson index for horse *i*of age* age*_*i*_ (binned in nine groups), *log(FEC* + *50)*_*i*_ is the horse transformed FEC (offset by 50 eggs/g, i.e. the minimal sensitivity of the measure), *Sex*_*i,j*_ is the horse sex (female, gelding or stallion) and *Farm*_*i,k*_ stands for horse_*i*_’s operation (eight levels) and was fitted as a random effect to account for the various drenching strategies. This analysis was restricted to the operations with a minimum of 10 horses from 156 and 32 horses from the Ukrainian and Polish datasets, respectively. The horse country of origin was not considered as it was partially confounded with horse age and accounted for by the farm effect. Difference in average horse ages between both populations was tested with a Student’s t-test. The model conditional* R*^2^ (accounting for the variance explained by both the random and fixed effects) was estimated with the ‘r.squaredGLMM()’ function of the MuMIn package. The percentage of variance explained by the fixed effects was estimated with the partR2 package [29].

### Impact of horse age on strongyle community diversity

To investigate how horse age was impacting on strongyle community structure, we re-analysed horse-wise community data gathered in Ukraine from 197 horses across 17 operations [10]. We restricted our analysis to eight farms with at least five age groups available, involving 112 individual horses (age range: 1– 22 years). Due to the paucity of data for some discrete age values, 8- and 9-year-old horses (*n* = 4 and *n* = 3 individuals, respectively) were binned together with the category of 7-year-old horses (*n* = 4 individuals), and horses aged > 10 years (16 horses between 11 and 22 years of age) were defined as a single age group. In all other categories, age groups corresponded to horse age. Ultimately, nine age groups were available for analysis, with at least 12 observations in each age group. Rare strongyle species (< 5% prevalence across the 112 horses; *n* = 2 species) were not considered further, leaving 31 strongyle species for inclusion in the analyses.

Because of this data curation, it was possible that past conclusions made on this dataset regarding the relationship between strongyle species richness and horse age [10] would differ from those in the present study. To ensure that data curation did not modify past conclusions, the species richness of horse *i* from Farm_*i,j*_ was modelled using the same analysis of variance as applied in the previous study [10]:

$${\mathrm{log}\,(Species\, richness) }_{i}\sim {\mathrm{log}(Faecal\, Egg\, Count)}_{i}+{Age }_{i}+{ Farm }_{i,j}$$

The relative contributions of the within-host diversity (ɑ-diversity) and the differences among communities (β-diversity) were estimated using an additive partitioning approach. In this framework, the γ-diversity (total diversity found across 8 farms in Ukraine) is expressed as the respective contributions of ɑ- and β-diversity terms [23]. This framework makes the comparison of ɑ- and β-diversity terms possible across the considered scales, including farms (which account for the anthelmintic management type), age group and individuals [24]. This analysis was implemented with the ‘adipart()’ function of the vegan package, as described in [30]. Parameter significance was determined following 1000 permutations [23]. This procedure shuffles species across samples while keeping their respective abundance and aggregation constant to determine the null distribution of ɑ- and β-diversity estimates at each hierarchical level. Females accounted for 75% of the dataset, and males (14 stallions and 13 geldings) were unequally represented across operations (6 in 2 farms, < 3 otherwise). To ensure that these results were not affected by the sex of the horse, the additive partitioning approach was applied twice: (i) either on the 112 horse datasets without considering their sex; or (ii) restricted to the 85 females. Because of the hierarchical nature of the analysis, ages were binned into discrete categories. To ensure that this transformation did not affect the conclusions drawn, age was fitted as a continuous predictor or as a categorical variable onto a non-metric multidimensional scaling (NMDS) ordination results (Bray–Curtis distance) applied to species counts using the ‘envfit()’ function of the vegan package [27].

Third, we searched for indicator species that would be more often encountered in a given age class. This analysis was achieved with the ‘multipatt()’ function of the indicspecies R package (v.1.7.9). We considered the indicator value (the relative mean species abundance in a group weighed by its relative frequency of occurrence in that group) [31] and the Pearson’s phi coefficient of association [32], whose significance was determined with 1000 permutations.

### Analysis at the anatomical niche level: species co-occurrences from necropsy data

To study the interactions between strongyle species within their hosts, we re-analysed individual necropsy report data collected in France [18]. Abundances of 20 strongyle species and other non-strongyle species (*Anoplocephala* sp*.*, *Oxyuris sp*.) were quantified within the caecum, ventral colon and dorsal-colon of 36 horses for the French dataset. These horses had not received anthelmintic drug within the last 2 months prior to necropsy despite being regularly treated [18], had their age registered and were infected by at least one parasite species. To avoid spurious signals associated with rare observations, we focused on the 15 species reaching at least 10% prevalence.

To support observations made in the French cohort, an independent dataset from Australia was considered. This dataset considered 150 horses and 29 species counted in the caecum, small colon or large colon. The data were restricted to 17 strongyle species with > 5% prevalence and 145 horses with at least one strongyle species identified. Other relevant metadata associated with each horse were not available (I Beveridge, personal communication).

To capture the strongyle community assembly pattern across anatomical niches, we applied the hierarchical Bayesian joint species distribution model [33, 34] implemented in the HMSC R package v.3.0–10 [34]. Joint species distribution models regress the whole vector of species abundances upon environmental predictors, thereby simultaneously accounting for the dependencies of species occurrences in a given sampling unit and for the effect of environmental covariates on these occurrences [35, 36]. This model offers an integrated framework to decompose species interactions into an environmentally dependent (raw co-occurrences) and residual co-occurrences [33, 37]. Raw co-occurrences would be compatible with shared response of two species to an environmental feature [38]. On the contrary, residual co-occurrences may arise from ecological interactions (facilitation or competition) or because of uncaptured variation [38]. We used the same approach as described elsewhere [39], fitting a first model accounting for the overall worm burden and a second full model accounting for the additional effects of the gut compartment (caecum, ventral colon and dorsal colon in the French dataset or caecum, small colon and large colon for the Australian dataset) and horse age for the French dataset as environmental covariates affecting species occurrences. In both cases, horse and horse × anatomical niche were fitted as random effects to account for inter-horse variation in species pairs co-occurrences and for residual variation not accounted for otherwise [39]. As such, these effects also account for unavailable metadata, for example anthelmintic treatment history.

To account for the over-dispersed nature of the count data, these two models were fitted using a hurdle-type approach considering either the log-transformed positive species counts or the binary presence or absence status using a probit-link function as described in [39]. The model predictive power was assessed by the *R*^2^ parameters (given by the HSMC package) for both species presence/absence data and species counts. For presence/absence modelling, receiver operating characteristic (ROC) curves (inferred using the pROC package) and area under the curve (AUC; estimated using the HSMC package) were considered. Parameters were estimated from 4000 posterior samples collected every 100 iterations from four Markov chain Monte Carlo (MCMC) chains. Each chain was run for 150,000 iterations, and a third of these were discarded as burn-in. Convergence was supported by effective sample sizes > 1000 and potential scale reduction showing little deviation from 1. Parameter significance with 95% posterior support was deemed significant.

## Results

### Relationship between species diversity and FEC at the horse level in Eastern Europe

More diverse strongyle communities may reflect reduced drug usage or more permissive hosts, and might be associated with higher parasite egg excretion. In line with this hypothesis, species richness and FEC were positively correlated in Poland (Spearman’s rank correlation coefficient, *rs* = 0.37, *P* = 0.04; *n* = 32) but not in Ukraine (*P* = 0.56; *n* = 156). On the contrary, the negative association between the Gini-Simpson index and FEC in the Ukrainian cohort (Spearman’s correlation coefficient, *rs* = − 0.2, *P* = 0.01; *n* = 156) did not apply in Poland (*P* = 0.34; *n* = 32).

**Fig. 1:**
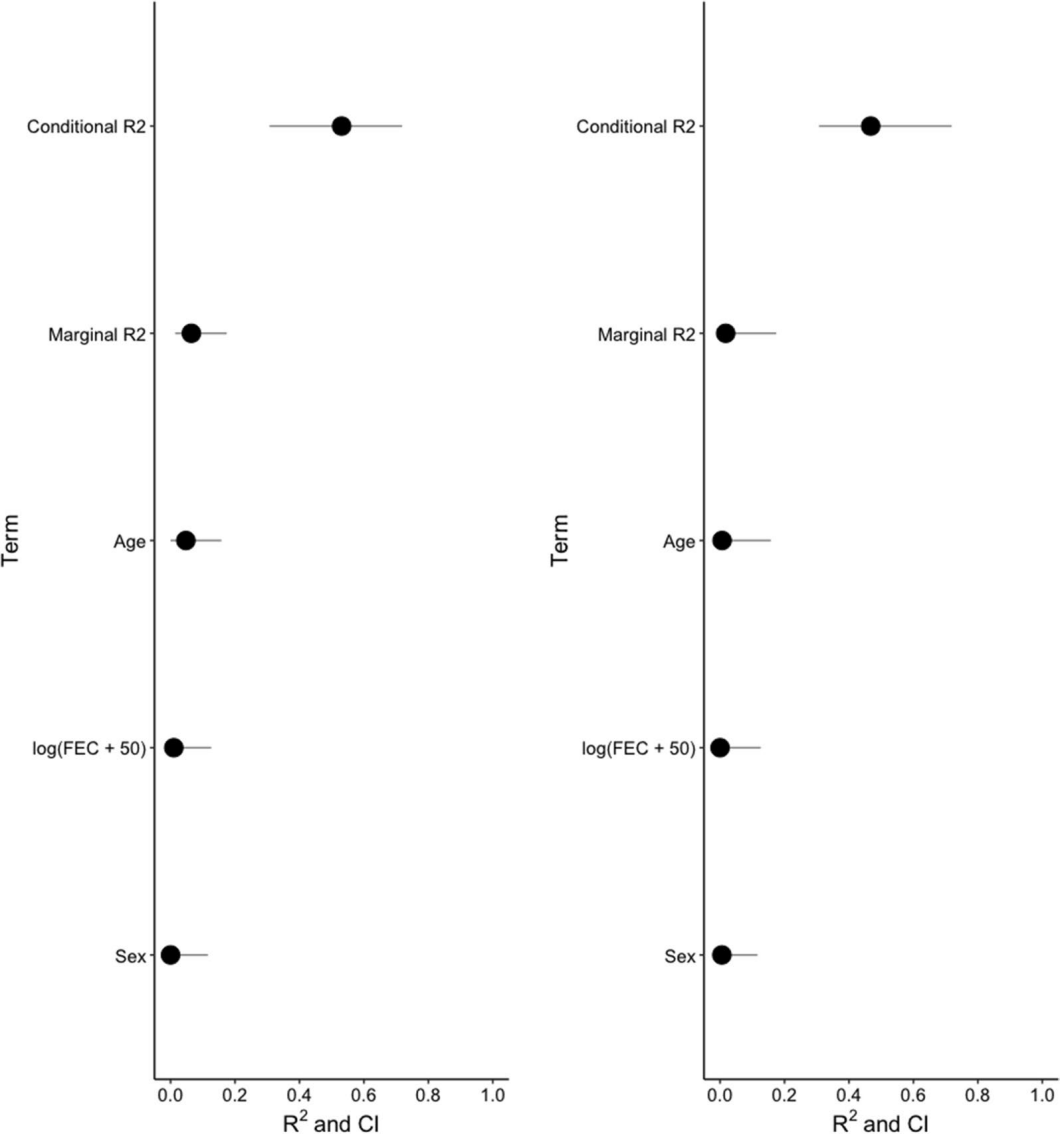
Partition of observed variation in equine strongyle community diversity across fixed effects. The explanatory power (*R*^2^) of the model and its associated fixed effects are plotted for species richness (left panel) and the Gini-Simpson index (right panel) of equine strongyle communities from Poland and Ukraine (*n* = 188 horses). The conditional *R*^2^ corresponds to the explanatory power of the full model with random effects, while the marginal *R*^2^ indicates the explanatory power of the fixed effects. In each case, a grey dot represents the estimated *R*^2^, and a horizontal line spans its associated confidence interval (CI). FEC, Fecal egg count

Because Ukrainian horses were significantly younger than their Polish counterparts (4.1 ± 4.6 vs 15.9 ± 5.4 years, respectively; Student’s *t*_(40)_ = 11, *P* < 0.0001), we further modelled each index to account for the respective contribution of horse’s age, FEC and other covariates (sex and operation). Linear modelling of community species richness or Gini-Simpson index explained 53.1% and 46.7% of the observed variance (Fig. 1; Additional file 1: Table S1). The variance explained by the fixed effects remained low in both cases, with marginal *R*^2^ of 6.5% and 1.7% for species richness and the Gini-Simpson index, respectively (Fig. 1; Additional file 1: Table S1), underscoring the importance of inter-operation variation in strongyle community diversity (Additional file 1: Table S1). Linear regression modelling found a significant negative association between horse age and the number of strongyle species recovered (*β*_age_ = − 0.14, standard error [SE] = 0.05; *t*_(176)_ = − 2.5, *P* = 0.01). This effect explained 4.76% of total variance in species richness (Fig. 1), thereby outweighing the 1.01% contributed by FEC (Fig. 1; *β*_FEC_ = 0.57, SE = 0.33; *t*_*(179)*_ = 1.72, *P* = 0.08). Of note, horse age was not correlated with worm burden (Spearman’s correlation coefficient, *rs* = 0.11, *P* = 0.1393; *n* = 188). Conversely, neither horse age nor horse FEC were significant predictors of the Gini-Simpson index (*t*_(173)_ = 1.44, *P* = 0.15), suggesting that variation in species dominance was little affected by FEC or the horse age in this population. The other considered fixed effects did not contribute significantly to the variance in species richness or Gini-Simpson index (Fig. 1; Additional file 1: Table S1).

### Contribution of horse age to strongyle community dissimilarity across Ukraine

To further establish the relative importance of horse age to the remodelling of strongyle communities, we applied a diversity partitioning framework to the Ukrainian dataset [10]. Because of the hierarchical nature of the analysis, nine age groups were considered (as described the Methods section), and data curation was applied to ensure a sufficient number of observations were available for each level of the factors of interest. We first checked that the previously reported relationship between strongyle species richness and horse age still held (Additional file 2: Table S2). Worm count was indeed inversely associated with horse age, i.e. worm count was higher in younger horses (*F*_(1, 102)_ = 10.2, *P* = 0.002; Additional file 2: Table S2). Significant variation was also found across the farms retained in the analysis (*F*_(7, 102)_ = 8.27, *P* < 0.0001). Therefore, the considered subset was taken to be a good representation of the original dataset [10]. Second, the differential effect of using age as a categorical predictor or a continuous predictor was tested. Following an NMDS ordination (Additional file 3: Figure S1), horse age had explanatory powers of 13.37% (*P* = 0.01) and 9.52% (*P* = 0.004) when considered as a categorical or a continuous predictor, respectively. This result suggests that horse age was a significant descriptor of the variation between communities irrespective of the applied binning.

**Fig. 2:**
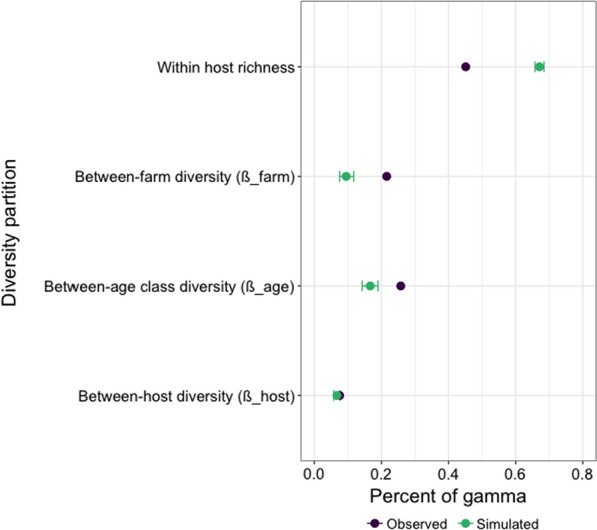
Partitioning of horse strongyle diversity across Ukraine operations. The regional diversity (γ-diversity) measured across 112 horses from 8 Ukrainian operations was partitioned into the within-host α-diversity and β-diversity found among groups of interest (host, age class and farm). For each diversity partition, the observed (purple dot) and expected values (obtained from 1000 simulations; green dot), with the 95% confidence interval shown as error bars, are expressed as the percentage of overall γ-diversity

Partitioning of overall γ-diversity across Ukraine operations revealed an outstanding contribution of within-horse α-diversity (45.1% of overall diversity) relative to the community dissimilarity coefficients (Fig. 2). However, the observed value was lower than the expected value under the null model (22.6% difference; Z-score = − 31.89, *P* = 0.001). Species diversity among horse age groups was the second most important contributor to γ-diversity (*β*_age_ = 25.8% of γ-diversity), and was significantly higher than that expected under a null model (Z-score = 7.54, *P* = 0.001; Fig. 2) and higher than that explained by between-farm variation (*β*_farm_ = 21.6% of γ-diversity; Fig. 2). Of note, the observed between-horse variation in species diversity did not depart from the simulated null distribution (0.08% difference; *P* = 0.12; Fig. 2). Restricting this analysis to the 85 females only yielded similar estimates although β-diversity between-farm (*β*_farm_ = 23.5% of γ-diversity) was slightly higher than β-diversity between horse age class (*β*_age_ = 22.5% of γ-diversity).

**Fig. 3:**
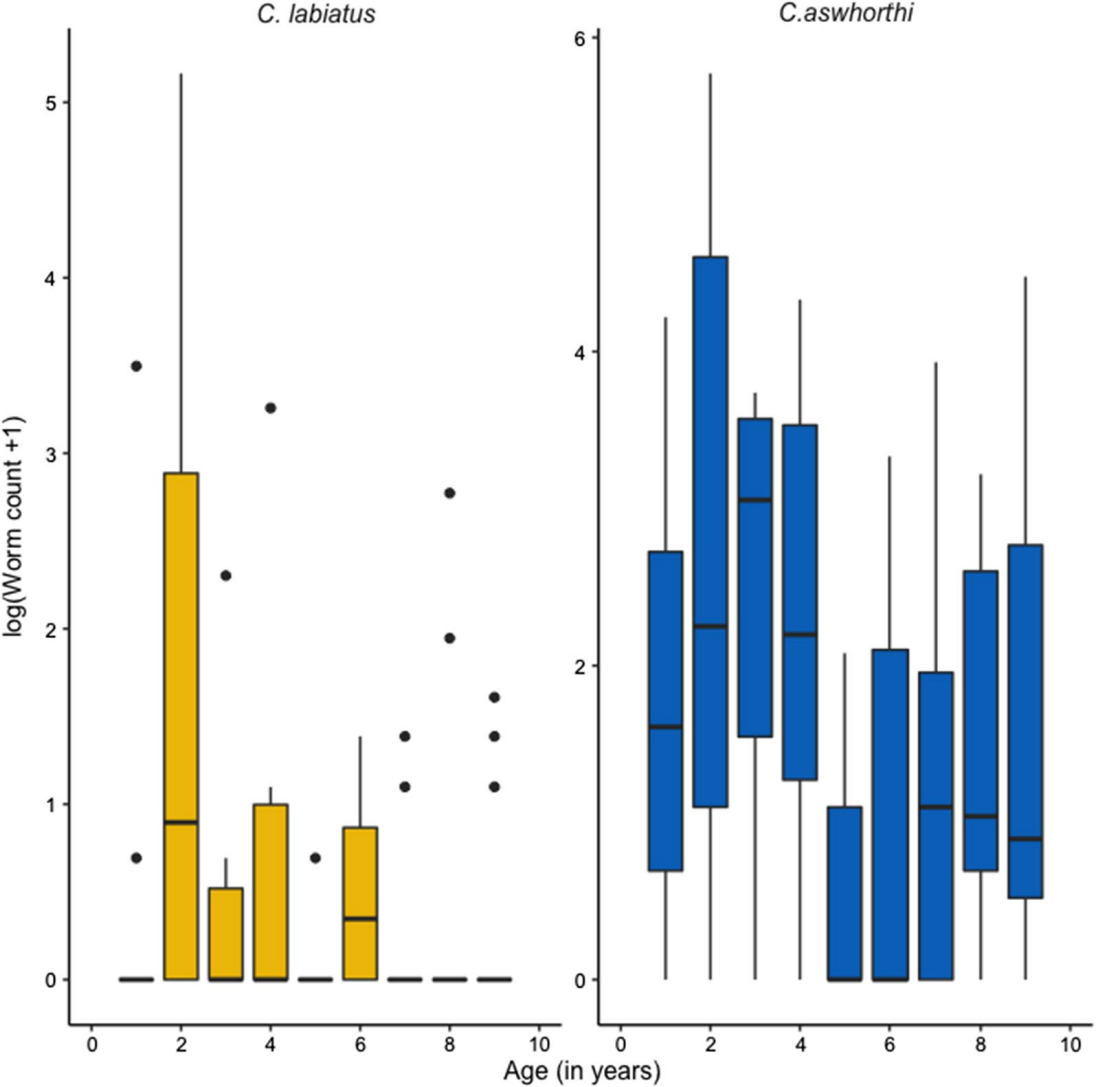
Presence of *Cylicocyclus ashworthi* and *Coronocyclus labiatus* are associated with younger horses. Each panel represents the distribution of log-transformed worm counts quantified after the deworming of horses of various ages in the Ukraine operations (total *n* = 112; *n* = 30, 12, 6, 10, 7, 8, 11, 12 and 16 for the respective nine age groups). Each box plot spans the inter-quartile range of each species count and the middle line represents the distribution median. Both species were predominant in 2-year-old horses

Due to the importance of age group as a driver of strongyle community structure, we also searched for indicator species that would preferentially be associated with a given age group. This analysis found that *Coronocyclus labiatus* and *Cylicocyclus ashworthi* were preferentially encountered in 2-year-old horses (Fig. 3), irrespective of the considered coefficient (*C. labiatus*: indicator value = 0.659, *P* = 0.007; *C. ashworthi*: indicator value = 0.585, *P* = 0.04; Pearson’s phi association coefficient = 0.42, *P* = 0.01 and 0.41, *P* = 0.01 for *C. labiatus* and *C. ashworthi,* respectively).

Altogether these results suggest that horse-level community diversity and species diversity variation between age classes are the major contributors driving measured diversity under the conditions encountered in the Ukraine operations.

### Co-occurrence of species within different gut compartments

**Fig. 4:**
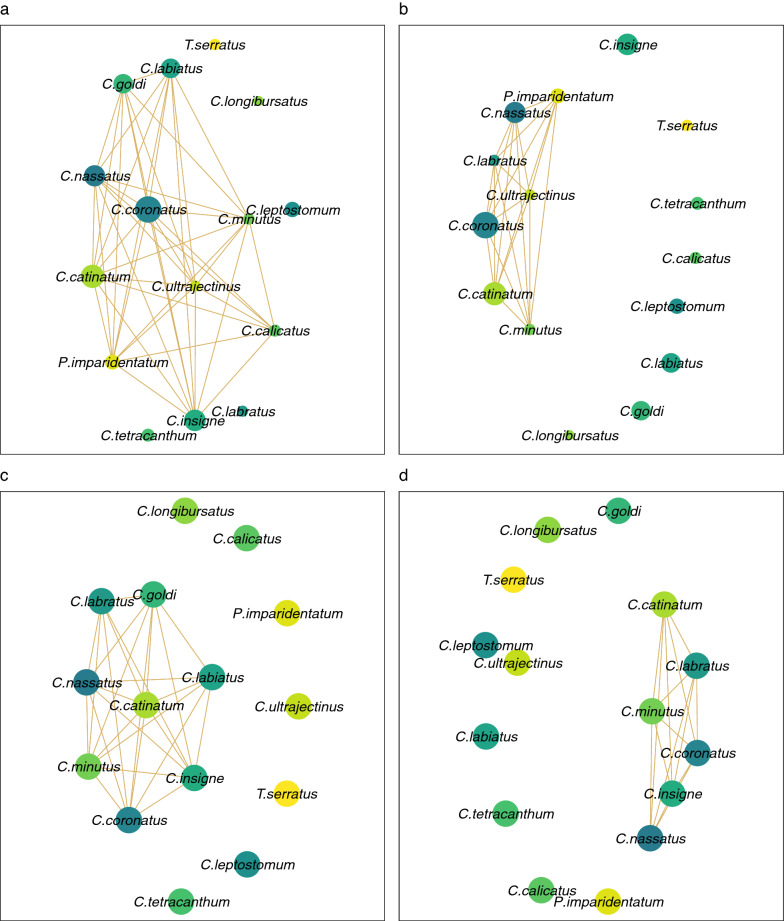
Strongyle species co-occurrence networks inferred from necropsy data collected in France. Networks represent species pair-wise association matrix estimated with the hierarchical species distribution model applied to worm counts collected in Normandy, France (*n* = 36 necropsied horses). Each species is represented as a node whose size is proportional to the species mean relative abundance (**c**, **d**) or mean prevalences (**a**, **b**) in the population. Edges indicate correlation with 95% posterior support (in occurrence for **a**, **b**; in abundance for **c**, **d**) with another species (pale brown if positive, green otherwise). The left panels (**a**, **c**) were drawn from the raw correlations and the right panels (**b**, **d**) were built from the residual correlation estimates obtained after accounting for variability in the total worm burden, horse age, anatomical niche and inter-horse variation. Node sizes are proportional to the species mean relative abundance (**c**, **d**) or mean prevalence (**a**, **b**) in the population

After establishing the relative contribution of horse age to the dissimilarity between strongyle communities, we undertook an analysis to model species interactions in the various gut compartments using data collected in France (Fig. 4). Observed patterns in this dataset were compared with co-occurrences from an Australian cohort of 141 horses (Additional file 4: Figure S2). The predictive power of the modelling approach was high, with a mean AUC of 0.90 and* R*^2^ of 0.79 for models based on presence-absence or positive abundance data, respectively (Additional file 5: Table S3; Additional file 6: Figure S3; Additional file 7: Figure S4, Additional file 8: Figure S5, Additional file 9: Figure S6). The gut compartment accounted for at least one quarter of the observed variation in species co-occurrences (Table 2), while inter-horse variation varied between 11.3 and 25% (Table 2).

**Table 2:** Proportion of variance explained by considered covariates in the residual co-occurrence models

Effect type	Covariate^a^	Normandy, France (*n* = 36 horses)		Victoria, Australia (*n* = 141 horses)	
		Abundance data (%)	Occurrence data (%)	Abundance data (%)	Occurrence data (%)
Fixed effect	Horse age	14.6	12.9	N/A	N/A
	Organ	31.2	23.8	61	48.3
	Worm burden	14.1	10.9	5.4	3.2
Random effect	Horse	12.8	19.1	11.3	26.5
	Horse × niche	27.3	33.4	22.3	22

Results are shown for two datasets collected in Australia [6] and France [19]

*N/A* Not available

^a^For each covariate of interest (fixed or random effect), the proportion of variance explained in strongyle species co-occurrence is given for the two model types considered using positive abundance data or presence-absence data

This host-related variance was higher with species occurrences than with their abundances, suggesting that the host acts as a filter for species establishment but has a more limited impact on their abundances. In line with the aforementioned effect of age on the dissimilarity between communities, horse age accounted for > 10% of species variance in the French cohort (Table 2).

The higher contribution of the worm burden in that cohort may reflect the fewer number of worms found in the French horses (75% quantile = 2040 worms) than in the Australian horses (75% quantile = 8266 worms).

Raw and residual co-occurrences only supported positive interactions between the considered strongyle species in the French and Australian populations (Fig. 4; Additional file 4: Figure S2; Additional file 10: Dataset 1), with the exception of a negative interaction (Spearman’s correlation coefficient *rs* = -0.49; Additional file 10: Dataset 1) between the occurrences *Coronocyclus coronatus* and *Cylicostephanus longibursatus* in the Australian populations (Additional file 4: Figure S2).

While raw co-occurrence coefficients were high across models and populations, estimates based on species presence/absence in the Australian population were milder between members of the *Cylicostephanus* genus (*C. calicatus*, *C. goldi* and *C. longibursatus*) and other species (Additional file 4: Figure S2).

The correction for environmental covariates however erased about half of observed co-occurrences in both abundance (11 out of 26 in the French data; 35 out of 81 in the Australian data) and presence-absence (21 out of 42 in the French data; 27 out of 98 in the Australian data) models (Fig. 4; Additional file 4: Figure S2). This pattern of generalized positive co-occurrence occurred for the most prevalent and abundant species and would support the shared response to the gut compartment, host age or overall infection intensity.

Of note, the presence of three *Cylicostephanus* species (*C. goldi* in the French horses, *C. calicatus* and *C. minutus* in the Australian cohort) was more dependent on inter-horse variation but not its abundance (Additional file 11: Figure S7; Additional file 12: Figure S8) suggesting limited expansion abilities once established in a host.

## Discussion

This study relied on data gathered in Europe and Australia to establish patterns of variation in strongyle community diversity across individual horses. The results suggest only a limited relationship between strongyle community diversity and their host FEC. On the contrary, they do underscore the importance of horse age on the diversity of strongylid communities and their remodelling. Lastly, the joint-distribution modelling of strongylid species defines a network of positive co-occurrences and identifies the relative contribution of environmental factors on species occurrence and abundance.

First, strongyle community diversity was mildly associated with the host parasite egg excretion in the considered cohorts from Eastern Europe, and variability was mostly accounted for by inter-operation variation. On the contrary, species richness decreased with increasing horse age, but with no evidence that dominance between strongyle species was affected in this process. Given the lack of a significant correlation between horse age and the collected worm burden, this result would be compatible with some cyathostomin species being more sensitive to the protective immune response progressively mounted by the host. Under this speculative perspective, the strongyle community would be reshaped over time with no association with FEC. The lack of a linear relationship between FEC and strongyle diversity may also mirror variations previously reported between strongyle species fecundities [14] or may reflect possible density dependence effects that are known to occur in trichostrongyle species infecting ruminants [40].

Our working hypothesis considered FEC to be an indicator of the host resistance potential. In horses, this would be similar to the ability to limit the parasite population fecundity rather than worm burden, as a poor correlation exists between strongyle burden and FEC in horses [11]. Under the tolerance perspective, i.e. the ability for damage control upon infection [41], higher FEC would be found in individuals able to cope with the presence of a fecund population. Such tolerance would be compatible with the mild modifications found in the blood of the infected host [42, 43] or in their gut microbiota whose stable state is not altered in the presence of significant egg excretion [43]. In this tolerance framework, the host would exert limited effects on the parasite community and, in turn, putative dilution effects of the most fecund parasites in more diverse parasite communities could contribute to obscure the relationship between observed FEC and parasite diversity. This situation has been observed in infected amphibians [44], whereby the relationship between virulence and parasite diversity depended on the qualitative composition of the parasite community and their infection dynamics [44].

Second, the regional diversity of strongyle communities sampled across the Ukraine was re-analysed to disentangle the relative contribution of horse age to the diversity of the strongyle communities. This analysis relied on age categories to both support the applied hierarchical framework and to provide sufficient observations for each category. While this binning may bias the results and prevent their generalization to other studies, the regression of horse age onto the NMDS ordination supported its significant contribution to β-diversity irrespective of the binning. The overall regional diversity was mostly explained by within-host richness and β-diversity across horse age groups and operations. Species richness is conditional upon the availability of host resources, leading to a higher parasite species richness in larger host animal species or for higher host density [45]. Species richness also depends on environment saturation by infective larvae [46]. The latter two aspects underscore the importance of pasture hygiene and limited stocking density for the management of strongyle infection in grazing livestock. Of note, saturation of the environmental contamination is usually expected to erase community dissimilarity among hosts [47], but we identified a significant remodelling of strongyle populations between age groups. This result is however in line with the significant association between species richness and horse age reported in the present study. This remodelling remains to be fully dissected to disentangle the respective effects of selection applied through the mounting of an effective immune response as horses age [15, 16, 48–50] and the drift arising from putative competitive processes between species. In this respect, the higher β-diversity across age groups may arise from priority effects between strongyle species that would counter-balance the effect of environmental saturation in eggs and infective larvae [47]. Such a priority effect would in turn yield a network of positive co-occurrences between species as found for mixed infection of rodents by malaria species [51].

Such a network of positive co-occurrences was found with the joint species modelling approach applied to the data collected from each gut compartment. The correction for environmental factors retained a core network of positive co-occurrences between four species, namely *Cyathostomum catinatum*, *Cylicocyclus nassatus*, *Cylicostephanus minutus* and *Coronocyclus coronatus*. These would be compatible with facilitative relationships between more divergent species that would be less prone to resource competition [52]. While the complexity of underpinning biological processes may obscure the observed co-occurrences patterns [53], these interactions were found across different parasite communities and with the lowest possible resolution, thereby supporting their biological meanings [54]. However, this may also indicate a similar response of these species to missing covariates, such as the season, past infections and treatment history, that were not accounted for because of missing data. In addition, the contribution of the resident gut microbiota may play a role in the definition of this particular structuring as significant interactions between microbial and helminth communities are known to occur in horses [55–57]. Specifically, the interactions between cyathostomin and protozoan species have long been suspected [5], and the results of a recent investigation suggest reciprocal benefits between the two taxonomic groups in susceptible Welsh ponies [55].

## Conclusion

Taken together, priority effects between equine strongyle species in young horses may contribute to enhancing the dissimilarity between strongyle communities across age groups and defining the network of positive co-occurrences between the most dominant genera. The simultaneous monitoring of both the microbial and parasite communities using the latest barcoding approaches [58] should provide valuable contributions to challenge these hypotheses.

## Supplementary Information

**Additional file 1: Table S1.** Linear regression modelling output of strongyle community diversity in Ukraine and Poland.

**Additional file 2: Table S2.** Linear regression modelling output of strongyle species count in Ukraine operations.

**Additional file 5: Table S3.** Predictive power of the co-occurrence modelling approach.

**Additional file 3: Figure S1.** Non-metric multidimensional scaling (NMDS; Bray-Curtis distance) ordination analysis of Ukrainian strongyle communities (*n* = 112 horses) recovered from eight operations.

**Additional file 4: Figure S2.** Strongyle species co-occurrence networks inferred from necropsy data collected in Victoria state, Australia (*n* = 141 horses).

**Additional file 6: Figure S3.** Species-specific receiver operating characteristic curve from the reduced model of species co-occurrence measured in Normandy, France.

**Additional file 7: Figure S4.** Species-specific receiver operating characteristic curve from the full model of species co-occurrence measured in Normandy, France.

**Additional file 8: Figure S5.** Species-specific receiver operating characteristic curve from the reduced model of species co-occurrence measured in Victoria State, Australia.

**Additional file 9: Figure S6.** Species-specific receiver operating characteristic curve from the full model of species co-occurrence measured in Victoria State, Australia.

**Additional file 11: Figure S7.** Species-specific variance decomposition across the factors of interest in the Australian horse cohort using species relative abundances (**a**) or presence/absence (**b**).

**Additional file 12: Figure S8.** Species-specific variance decomposition across the factors of interest in the French horse cohort using species relative abundances (**a**) or presence/absence (**b**).

**Additional file 10. Dataset 1.** Strongyle species co-occurrence estimates derived from data collected in Normandy, France or Victoria State, Australia.

## Data Availability

The R scripts used for analysis under the following repository (https://github.com/guiSalle/EquineStrongylidCommunity). The associated raw data have been deposited under the INRAE data repository (https://doi.org/10.57745/FQ7RUA).
